# MsDpo4—a DinB Homolog from *Mycobacterium smegmatis*—Is an Error-Prone DNA Polymerase That Can Promote G:T and T:G Mismatches

**DOI:** 10.1155/2012/285481

**Published:** 2012-03-15

**Authors:** Amit Sharma, Deepak T. Nair

**Affiliations:** National Centre for Biological Sciences (NCBS-TIFR), GKVK Campus, Bellary Road, Bangalore 560065, India

## Abstract

Error-prone DNA synthesis in prokaryotes imparts plasticity to the genome to allow for evolution in unfavorable environmental conditions, and this phenomenon is termed adaptive mutagenesis. At a molecular level, adaptive mutagenesis is mediated by upregulating the expression of specialized error-prone DNA polymerases that generally belong to the Y-family, such as the polypeptide product of the *dinB* gene in case of *E. coli*. However, unlike *E. coli*, it has been seen that expression of the homologs of *dinB* in *Mycobacterium tuberculosis* are not upregulated under conditions of stress. These studies suggest that DinB homologs in *Mycobacteria* might not be able to promote mismatches and participate in adaptive mutagenesis. We show that a representative homolog from *Mycobacterium smegmatis* (MsDpo4) can carry out template-dependent nucleotide incorporation and therefore is a DNA polymerase. In addition, it is seen that MsDpo4 is also capable of misincorporation with a significant ability to promote G:T and T:G mismatches. The frequency of misincorporation for these two mismatches is similar to that exhibited by archaeal and prokaryotic homologs. Overall, our data show that MsDpo4 has the capacity to facilitate transition mutations and can potentially impart plasticity to the genome.

## 1. Introduction

For proper cellular functioning, the integrity of the genome has to be maintained and it is essential that replication should be error free. Replicative DNA Polymerases carry out template-dependent synthesis of DNA with high fidelity. Any errors that might appear during replication are corrected by the mismatch repair system [1]. However, under conditions of stress, increase in the frequency of appearance of heritable mutations can allow the organism to adapt to the environment and relieve selection pressure [2–4]. This phenomenon is termed adaptive mutagenesis. It has been suggested that pathogenic bacterial strains utilize adaptive mutagenesis to develop resistance against therapeutic agents [4–6]. Studies in the past decade on prokaryotes have shown that in an adverse environment, adaptive mutagenesis is mediated by expression of error-prone DNA polymerases [3, 7]. These specialized DNA polymerases, usually classified in the Y-family, appear to possess distinct active sites that allow them to accommodate non-Watson-Crick base pairs and thus promote mismatches [8, 9]. These enzymes generally exhibit low fidelity and low processivity, and these properties are exploited to facilitate adaptive mutagenesis.

In *E. coli*, it has been shown that DNA polymerases IV (EcDpo4/DinB) and V (EcDpo5/UmuCD_2_') are responsible for stress-induced mutagenesis [2, 3]. Orthologs of these two enzymes have also been implicated in mutagenesis in other organisms [3]. Mutant strains of *E. coli* lacking these polymerases exhibit substantial reduction in fitness during starvation conditions as compared to wild-type strains [10]. Unlike EcDpo5, EcDpo4 (encoded by the *dinb* gene) is a single-chain polypeptide, and genetic studies probing for the reversion of a frameshift mutation in the *lac* promoter have shown that EcDpo4 is responsible for 85% of adaptive point mutations during the stationary phase [11]. Additionally, it has been shown recently that expression of EcDpo4 is sufficient for stress-induced mutagenesis [12].

Orthologs of EcDpo4 have been identified in other pathogenic and nonpathogenic bacterial strains such as *Mycobacterium tuberculosis* (*Mtb*) and *Mycobacterium smegmatis* (*Msm*), respectively. *Mtb* possesses two putative EcDpo4 homologs, DinB1 (Rv1537) and DinB2 (Rv3056). It was seen that the Y-family DNA polymerases are not upregulated on stress-induced by mitomycin treatment [13]. Boshoff and colleagues had shown that treatment of *Mtb* cells with DNA damaging agents leads to the upregulation of the C-family DNA polymerase DNAE2 (Rv3370c) and there is no significant change in levels of DinB1 and DinB2 [14]. These studies implied that DNAE2 is the primary enzyme responsible for adaptive mutagenesis in case of *Mtb *[14]. Additionally, genetic studies probing for the effect of loss of function of DinB homologs in *Mtb* showed no significant changes in the phenotype [15]. This study also showed that there was no increase in the frequency of mutations when these homologs were expressed ectopically in *Msm*, and the authors suggest that mycobacterial DinB homologs function differently from those in other bacteria. The authors mention that attempts to clone and express DinB1 and DinB2 were met with limited success. These studies raise the possibility that mycobacterial homologs of Y-family DNA polymerases might not be able to promote mismatches. To discern the biochemical potential of EcDpo4 homologs from *mycobacteria*, we have carried out studies on one representative of this group from *Msm*.

The genome sequence of *Msm* shows the presence of three putative EcDpo4 homologs. These genes are annotated as *msmeg_1014*, *msmeg_3172*, and* msmeg_6443* in the KEGG Database. Sequence comparison of MSMEG_1014, with that of EcDpo4 and DinB homologs from other organisms, shows there is complete conservation of the key residues necessary for DNA polymerase activity. In this paper, we demonstrate that the polypeptide product of *msmeg_1014* (designated herein as MsDpo4) is a DNA polymerase. We show that MsDpo4 is able to carry out template-dependent nucleotide incorporation and mutation of one of the predicted active site residues leads to loss of this activity. In addition, the fidelity profile of MsDpo4 shows that this enzyme has the ability to promote mismatches on undamaged DNA. Steady-state kinetic analysis shows that MsDpo4 exhibits a slightly heightened ability for promoting G:T and T:G mismatches compared to known prokaryotic and archaeal DinB homologs. Overall, MsDpo4 has the ability to increase the frequency of mutations and thus can potentially participate in the initial steps of adaptive mutagenesis.

## 2. Materials and Methods

### 2.1. Site-Directed Mutagenesis

The *msmeg_1014* gene was cloned into bacterial expression vector pGEX6P1 (GE Healthcare) as mentioned elsewhere [16]. The clone (PGEX6P1_1014) will give rise to a fusion polypeptide of GST-MsDpo4 on recombinant expression and was used for further studies.

To prepare the MsDpo4D107A protein where Asp107 was mutated to Ala, the *msmeg_1014* gene was mutated using the QuickChange site-directed mutagenesis kit (Stratagene). Oligonucleotide primers (forward primer 5′-AGGTGTGGGGCTGG**GCC**GCGGCGTATCTGGGCGC-3′ and reverse primer 5′-GCGCCCAGATACGCCGC**GGC**CCAGCCCCACACCT-3′) designed for this purpose substituted the triplet codon for Ala (5′-gcc-3′ in forward primer and 5′-ggc-3′ in reverse primer) in place of that for Asp (5′-gac-3′). A clone (PGEX6P1_m1014) that showed the presence of the correct mutation (D107 to A) by sequencing was used for further studies. The native and mutant (MsDpo4D107A) proteins were purified using a protocol mentioned in detail elsewhere [16]. The final yield of MsDpo4D107A was ~3 mgs (from 5 litres of bacterial culture), and the protein was concentrated to a final concentration of 1.7 mM.

### 2.2. Primer Extension Assay

The use of oligonucleotides with a fluorescent label has been shown to be a reliable alternative to radiolabelling [17]. For primer extension assays, primer oligonucleotide (P1) with a 5′ 6-FAM label was purchased from the Keck Centre (Yale University). Four template oligonucleotides (T1, T2, T3, T4) were purchased from Sigma Genosys. T1, T2, T3, and T4 were designed to present four different nucleotides G, T, C, and A, respectively, at the templating position in the active site (Table 1). DNA substrates T1P1, T2P1, T3P1, and T4P1 prepared by annealing the primer P1 (5′6-FAM- CGTACTCGTAGGCAT 3′) with each of the four 50-mer templates T1 (TCCTACCGTGCCTACCTGAACAGCTGGTCACACT**G** ATGCCTACGAGTACG), T2 (TCCTACCGTGCCTACCTGAACAGCTGGTCACACA**T**ATGCCTACGAGTACG), T3 (TCCTACCGTGCCTACCTGAACAGCTGGTCACACA**C**ATGCCTACGAGTACG) and T4 (TCCTACCGTGCCTACCTGAACAGCTGGTCACACG**A**ATGCCTACGAGTACG), respectively. In addition, the labeled primer oligonucleotide was annealed with the oligonucleotide B1 (5′ ATGCCTACGAGTACG 3′) to generate a blunt-ended DNA substrate. For primer extension assay, the reaction mixture (20 *μ*L) consisted of 25 *μ*M of all or one dNTP, 100 nM of DNA substrate, 0.1 mM ammonium sulfate, 2.5 mM MgCl_2,_ 4 *μ*L of 5X assay buffer (125 mM Tris-Cl pH = 8.0 and 5 mM DTT), and 50 nM of MsDpo4 (wild type or mutant). In case of the blunt-ended substrate (B1P1), the DNA concentration was 20 nM and two different enzyme concentrations of 20 nM and 50 nM were added to a reaction mixture containing all dNTPs. After incubation for 2 hours at 37°C, all reactions were terminated by addition of 10 *μ*L of stop solution (80% formamide, 1 mg/mL Xylene C, 1 mg/mL bromophenol blue, and 20 mM EDTA) followed by 2 mins of incubation at 95°C. This mixture was immediately transferred to ice for 10 mins and was subsequently loaded onto a 20% polyacrylamide gel containing 8 M urea and 1X TBE. Resolved reaction products on the gel were observed by excitation at 488 nm, and the bands were visualized and recorded using a Typhoon scanner (GE Healthcare). The sensitivity of this experimental system was such that a band could be detected for a sample containing 0.15 pmol of labeled oligonucleotide. The intensity of the observed bands was quantified using Image Quant TL, 1D gel analysis software. The level of incorporation for each reaction was calculated using the following equation:

(1) $$\text{Percentage}\;\text{incorporation}\;=\;\frac{Is}{\left(Is\;+\;Ip\right)}\times 100,$$

where *Is* = intensity of band that has shifted upwards due to incorporation, *Ip* = intensity of the nonshifted primer band in the same lane.

### 2.3. Steady-State Kinetic Analysis

For steady-state kinetic analysis, the reaction has to be terminated when it is in the linear range and hence initially time-course reactions were carried out to establish a time point at which not more than 20% of the primer had been extended [18, 19]. These reactions were carried out for the template nucleotide: incoming nucleotide combinations (i) dG:dCTP, (ii) dG:dTTP, (iii) dT:dATP, and (iv) dT:dGTP. Once the correct time of incubation was standardized, multiple reactions were carried out for each combination with varying concentration of incoming nucleotide. In case of dG:dCTP and dT:dATP, the concentration of the incoming nucleotide was varied from 1 to 100 *μ*M. In case of dG:dTTP and dT:dGTP, the concentration of the incoming nucleotide was varied from 10 to 5000 *μ*M. All reactions were initiated by the addition of incoming nucleotide, and the products were resolved on a 20% polyacrylamide gel containing 8 M urea. The resolved products on the gel were observed by excitation at 488 nm, and the bands were visualized and recorded using Typhoon scanner (GE Healthcare). The intensity of the observed bands was quantified using Image Quant TL, 1D gel analysis software. Apparent *K*_*m*_ and *V*_max⁡_ values for each combination of template and incoming nucleotide were calculated using a Lineweaver-Burk plot [19]. A representative analysis (for template G and incoming dCTP) is shown in Supplementary Figure 1 (see Supplementary Material available online at doi:10.1155/2012/285481). The ratio of *V*_max⁡_/*K*_*m*_ is equal to the catalytic efficiency and the frequency of misincorporation was calculated as follows:

(2) $$f_{\text{inc}}=\;\frac{\left[V_{\max}/K_{m}\;\left(\text{for}\;\text{misincorporation}\right)\right]}{\left[V_{\max}/K_{m}\;\left(\text{for}\;\text{correct}\;\text{incorporation}\right)\right]}.$$

## 3. Results

### 3.1. Sequence Analysis and Site-Directed Mutagenesis

**Figure 1:**
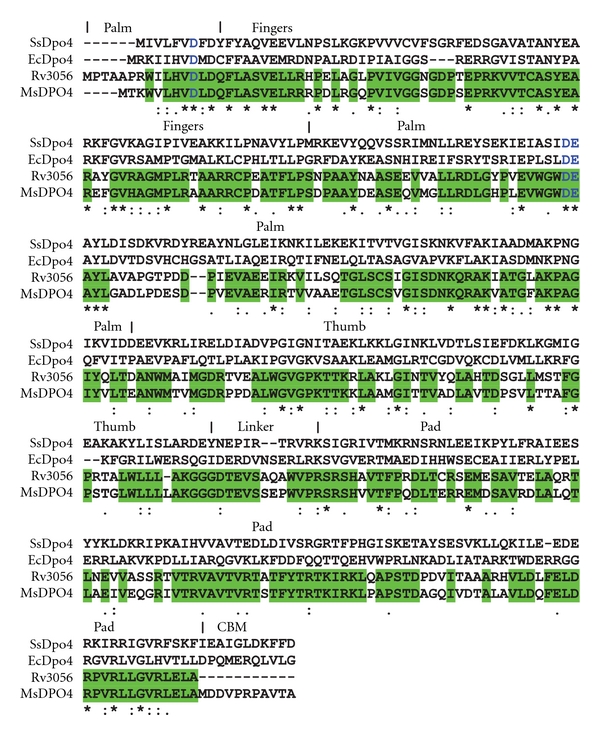
Sequence alignment of representative DinB homologs. The sequences of Dpo4 from *Sulfolobus solfataricus *(SsDpo4), *Escherichia coli *(EcCpo4), *Mycobacterium tuberculosis* (Rv3056), and *Mycobacterium smegmatis *(MsDpo4) are compared. Active site residues are displayed in blue color. The identical residues in Rv3056 and MsDpo4 are displayed with similar background colour. The different domains (palm, fingers, thumb, and PAD) observed in Y-family DNA polymerases are delineated on the basis of the ssDpo4 structure (1JX4). CBM represents the processivity clamp binding motif.

 In the KEGG database, there are three genes from *Msm* that are predicted to belong to the Y-family of DNA polymerases. MsDpo4 was of near similar length and showed substantial homology to DinB from *Escherichia coli* (EcDpo4) and also to the structurally well-characterized archaeal enzyme SsDpo4 from *Sulfolobus solfataricus*. The sequence of MsDpo4 showed the presence of the DEAY motif that is critical for DNA polymerase activity [20, 21]. Analysis of the MsDpo4 in the context of the available ssDpo4 structure (PDB code: 1JX4) revealed that D9, D107, and E108 should be the three residues critical for catalysis (Figure 1). A possible catalytic mutant of MsDpo4 wherein the D107 was mutated to alanine (MsDpo4D107A) was therefore prepared by site-directed mutagenesis of the wild-type gene, and the mutant protein was purified.

Comparison with sequences of predicted members of the Y-family in the *Mtb* genome revealed that MsDpo4 shows greater homology (71% identity) to Rv3056 (Figure 1). The catalytic residues are conserved, and one important difference was observed. MsDpo4 has an extra 13 residues at the C-terminus, and this region could correspond to the processivity clamp binding motif [22, 23].

### 3.2. MsDpo4 Exhibits DNA Polymerase Activity

**Figure 2:**
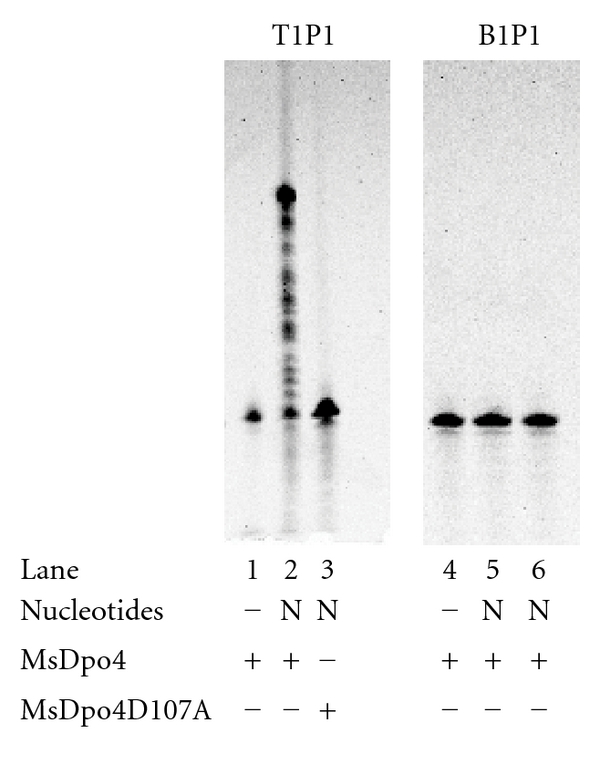
DNA polymerase activity of MsDpo4. The results for primer extension assay in case of wild-type and mutant (D107A) MsDpo4 are displayed (lanes 1–3). The results for assays with blunt ended substrate and wild-type MsDpo4 at two different enzyme concentrations (lane 5–20 nM; lane 6–50 nM) are also displayed.

**Figure 3:**
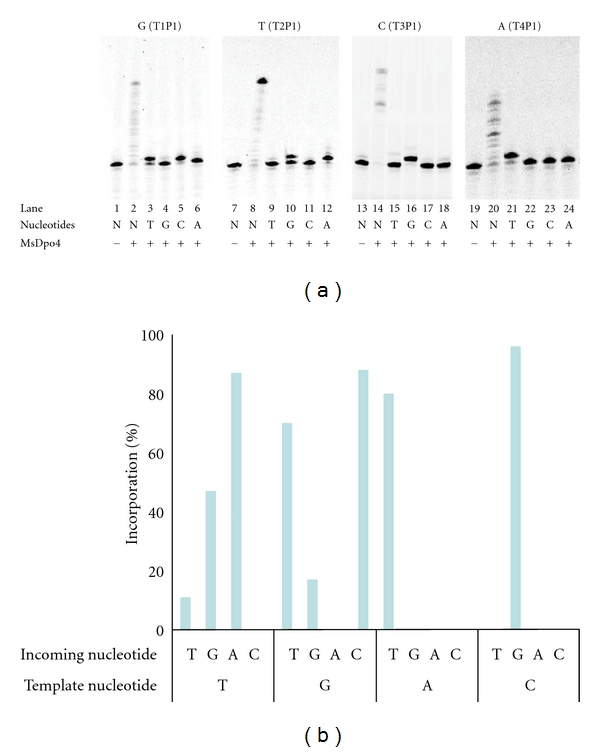
Fidelity profile of MsDpo4. (a) The results for primer extension assay wherein MsDpo4 was incubated with DNA duplexes (T1P1, T2P1, T3P1, and T4P1) that presented different template nucleotides in the active site (G, T, C, and A) are displayed. This mixture of MsDpo4 and DNA either included all deoxynucleotide triphosphates (denoted as N) or the four different dNTPs separately (G, T, C, and A). (b) Bar diagram exhibiting the incorporation profile of MsDpo4. Although the Watson-Crick base pairing is the preferred mode of nucleotide selection, MsDpo4 does exhibit significant ability to promote G:T and T:G mismatches.

To check if MsDpo4 shows polymerase activity, primer extension assays were carried out. The DNA substrate T1P1 was prepared by annealing 50-mer template (that presented G in the templating position) and labeled (5′ 6-FAM) 15-mer primer. In the presence of all dNTPs, MsDpo4 was able to extend the primer (Figure 2, lane 2; Figure 3(a) lane 2) and therefore should possess DNA polymerase activity. MsDpo4D107A where Asp107 was mutated to Ala was purified using same method as used for MsDpo4. In contrast to the wild-type protein, MsDpo4D107A was unable to extend the primer (Figure 2, Lane 3). The fact that mutating one of the catalytic Asp residues to Ala renders the preparation devoid of DNA polymerase activity discounts the possibility that the observed activity could be due to trace amounts of a contaminating polymerase. MsDpo4 was unable to add any nucleotides to the blunt-ended substrate (B1P1) even at high enzyme concentrations and therefore is not a deoxynucleotidyl transferase (Figure 2, lanes 4, 5, and 6). The primer extension for MsDpo4 was repeated with three other substrate DNAs (T2P1, T3P1, and T4P1) that presented T, C, and A in the templating position. In the presence of all four nucleotides, the enzyme was able to extend the primer in all three cases (Figure 3(a), lanes 8, 14, and 20).

### 3.3. Fidelity Profile of MsDpo4

To check fidelity of MsDpo4 in synthesizing DNA, primer extension assays were conducted with the four different DNA substrates (T1P1, T2P1, T3P1, and T4P1) in presence of only one deoxynucleoside triphosphate (dNTP) at a time. MsDpo4 incorporated correct nucleotides opposite template nucleotide for all the four DNA substrates (Figure 3(a), lanes 5, 12, 16, and 21). However, misincorporations of nucleotides that do not correspond to the Watson-Crick base pairing scheme were also observed for some combinations of template and incoming nucleotides (Figure 3(a)). MsDpo4 appears to exhibit substantial ability to incorporate T opposite G (Figure 3(a), lane 3) and G opposite T (Figure 3(a), lane 10). In addition minor amounts of product are also formed for G opposite G (Figure 3(a), lane 4) and T opposite T (Figure 3(a), lane 9). The level of incorporation was quantitated in terms of the percentage of primer that is extended in the presence of nucleotide. The observed percentages are plotted in the form of a bar diagram (Figure 3(b)), and it is clear that MsDpo4 can promote G:T and T:G mismatches.

Steady-state kinetic analysis were carried out for the G:T and T:G misincorporations. In order to be able to calculate the frequency of misincorporation of these combinations of templates and incoming nucleotides, similar analysis was also carried out for G:C and T:A incorporations. The reactions were carried out with 1 nM of enzyme and 20 nM of substrate DNA in case of G:C and T:A pairs and 10 nM of enzyme and 20 nM of substrate in case of the G:T and T:G pairs with different concentrations of incoming nucleotide. The apparent *K*_*m*_ values for the incoming nucleotide, *V*_max⁡_/*K*_*m*_, and the frequency of incorporation are shown in Table 2. The misincorporation frequencies for G:T and T:G are 2.1 × 10^−4^ and 2.5 × 10^−4^ and are slightly lower than those reported in case of ssDpo4 and EcDpo4/DinB for these mismatches [24–26]. The results obtained from kinetic analysis clearly show that, although Watson-Crick base pairing is the preferred mode of nucleotide selection, MsDpo4 does possess significant ability to promote G:T and T:G mismatches.

## 4. Discussion

Genetic studies have implicated *dinD* homologs in stress-induced mutagenesis in case of *Escherichia coli*, *Bacillus subtilis*, *Pseudomonas aeruginosa*, and *Pseudomonas putida *[2, 3, 27–29]. Among these orthologs, only EcDpo4 protein from *E. coli* has been subjected to a rigorous biochemical characterization [26, 30]. The study of stress-induced mutagenesis is especially important in mycobacteria because pathogenic strains such as *Mtb* undergo continuous evolution resulting in the appearance of resistance to a variety of therapeutic agents [31, 32]. Genetic studies on *Mtb* strains have suggested that error-prone DNA polymerases belonging to the Y-family (DinB1 and DinB2) play no role in mutagenesis. It was shown that perturbation of the function of DinB1 and DinB2 had no discernable effect on the phenotype. These studies hint that representatives of the Y-family in mycobacteria might not be able to promote mismatches.

Our *in vitro* studies clearly show that one of the representatives from *Msm*, MsDpo4 (that exhibits high homology to DinB2), is enzymatically active and proficient in carrying out template-dependent nucleotide incorporation. Studies examining the fidelity of MsDpo4 show that although this enzyme prefers Watson-Crick mode of base pairing, it exhibits substantial ability to promote dG:dTTP and dT:dGTP mismatches with nearly identical catalytic efficiency. Unlike EcDpo4 and the archaeal homolog ssDpo4, the ability of MsDpo4 to promote mismatches appears to be restricted mostly to G:dTTP and T:dGTP mismatches. Structures of ternary complexes of DNA polymerases with DNA and nucleotides that lead to the presence of a mismatch in the active site have been determined earlier [8, 33–38]. These studies show that the manner in which a mismatch is accommodated in the active site appears to be unique for each polymerase and also depends on which nucleotide is present at the templating and incoming positions. Initially it was believed that G:T mismatch will generally occupy a Wobble configuration. However, structural studies in the last decade have shown that G:T mismatches can be stabilized in the active site not only through wobble base pairing but also through the following configurations: (i) reverse wobble, (ii) Hoogsteen (T occupies a syn conformation), and (iii) canonical Watson-Crick base pair [8, 9, 33, 34, 36, 37]. Further studies are required to determine the mode of base pairing utilized by MsDpo4 to stabilize the G:T and T:G mismatches. Structural studies on other members of the Y-family have shown that the incoming nucleotide is locked into position through interactions between protein residues and the sugar and triphosphate moiety of the incoming nucleotide [39–43]. Hence, only the template nucleotide will have conformational flexibility to facilitate a mode of base pairing with the incoming nucleotide that can enable catalysis. Therefore, it is possible that MsDpo4 employs different modes of base pairing to stabilize dG:dTTP and dT:dGTP base pairs in its active site.

The ability of MsDpo4 to promote G:T and T:G mismatches will ultimately lead to transition mutations G to A and T to C in genomic DNA. In addition to G:T mismatches, this enzyme can misincorporate, to a minor extent, T opposite T and G opposite G. These mismatches will lead to transversion mutations after another round of replication. Since *Mycobacteria* do not possess a mismatch repair system, once recruited to the replication fork the activity of MsDpo4 can lead to the appearance of heritable mutations. Overall, this representative of the Y-family of DNA polymerases from *Mycobacteria* is capable of carrying out both accurate and error-prone synthesis of DNA. The observed fidelity profile of MsDpo4 implies that this enzyme has the biochemical capacity to participate in the initial steps of adaptive mutagenesis and play a role in imparting plasticity to the genome to survive adverse environmental conditions.

MsDpo4 shows very high similarity (74% identity) with Rv3056 (DinB2). In addition, orthologs of EcDpo4/DinB from other pathogenic strains of mycobacteria *M. intracellulare* and *M. avium* also show high homology (~80% identity) with MsDpo4 (see Supplementary Figure 2 in the Supplementary Material available online at doi: 10.1155/2012/285481). It is therefore possible that there will be substantial overlap in the biochemical properties of these enzymes. This suggests that once recruited to the replication fork, these proteins can give rise to mutations in the genome. In case of *Mtb*, the appearance of point mutations in certain genes has been shown to render resistance to different therapeutic antibiotics [31, 32]. Overall, due to their predicted ability to promote mutations, the orthologs of MsDpo4 in pathogenic strains might play an important role in the appearance of resistance to therapeutic antibiotics.

## Supplementary Material

Supplementary Material includes a representative steady state kinetic data analysis and a comparison of the sequences of MsDpo4 homologs from Mycobacteria.

## Figures and Tables

**Table 1:** 

Oligonucleotides	
T1	5′ TCCTACCGTGCCTACCTGAACAGCTGGTCACACT**G**ATGCCTACGAGTACG 3′
T2	5′ TCCTACCGTGCCTACCTGAACAGCTGGTCACACA**T**ATGCCTACGAGTACG 3′
T3	5′ TCCTACCGTGCCTACCTGAACAGCTGGTCACACA**C**ATGCCTACGAGTACG 3′
T4	5′ TCCTACCGTGCCTACCTGAACAGCTGGTCACACG**A**ATGCCTACGAGTACG 3′
P1	5′ CGTACTCGTAGGCAT 3′
B1	5′ ATGCCTACGAGTACG 3′

Substrate duplexes	

T1P1 (G)	5′ TCCTACCGTGCCTACCTGAACAGCTGGTCACACT**G**ATGCCTACGAGTACG 3′
	3′ TACGGATGCTCATGC 5′
T2P1 (T)	5′ TCCTACCGTGCCTACCTGAACAGCTGGTCACACA**T**ATGCCTACGAGTACG 3′
	3′ TACGGATGCTCATGC 5′
T3P1 (C)	5′ TCCTACCGTGCCTACCTGAACAGCTGGTCACACA**C**ATGCCTACGAGTACG 3′
	3′ TACGGATGCTCATGC 5′
T4P1 (A)	5′ TCCTACCGTGCCTACCTGAACAGCTGGTCACACG**A**ATGCCTACGAGTACG 3′
	3′ TACGGATGCTCATGC 5′
B1P1 (blunt)	5′ ATGCCTACGAGTACG 3′
	3′ TACGGATGCTCATGC 5′

**Table 2:** Frequency of dG:dTTP and dT:dGTP misincorporation by MsDpo4 as determined by steady-state analysis.

Template: incoming nucleotide	*K*_*m*_ (*μ*M)	*V*_max⁡_/*K*_*m*_(nM of enzyme^−1^ min^−1^)	*f*_inc_
dG:dCTP [4]	4.3 (0.69)	16.16 (5.6)	1
dG:dTTP [4]	165.5 (39.70)	3.34 × 10^−3^ (7.4 × 10^−4^)	2.1 × 10^−4^
dT:dATP [4]	5.5 (1.54)	12.74 (2.3)	1
dT:dGTP [4]	166.5 (39.01)	3.2 × 10^−3^ (8.1 × 10^−4^)	2.5 × 10^−4^

The nucleotide misincorporation ratio, *f*_inc_ = (*V*_max⁡_/*K*_*m*_)_misincorporation_/(*V*_max⁡_/*K*_*m*_)_correct  incorporation_. For example, *f*_inc(GT)  _= (*V*_max⁡_/*K*_*m*_)_GT_/(*V*_max⁡_/*K*_*m*_)_GC_. Number of experimental trials are given in [].

Standard errors between the experiments are given in ().
