# Supplementary figures and images for: MsDpo4—a DinB Homolog from Mycobacterium smegmatis—Is an Error-Prone DNA Polymerase That Can Promote G:T and T:G Mismatches
Source: J Nucleic Acids. 2012 Mar 15;2012:285481. doi: 10.1155/2012/285481 (PMC3317225; doi:10.1155/2012/285481)

A.

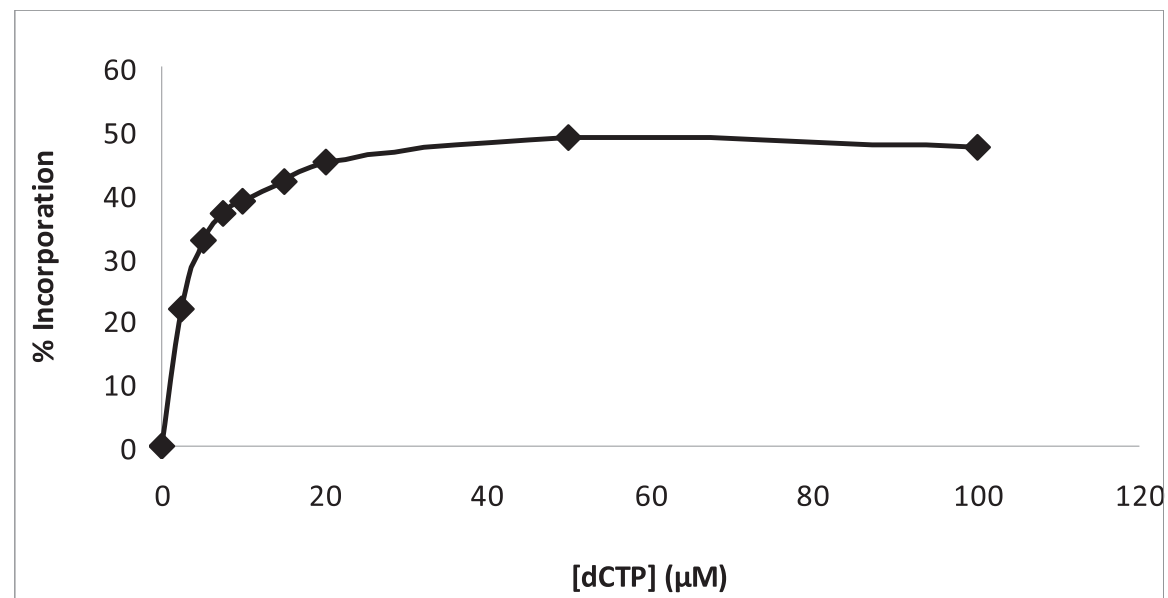

B.

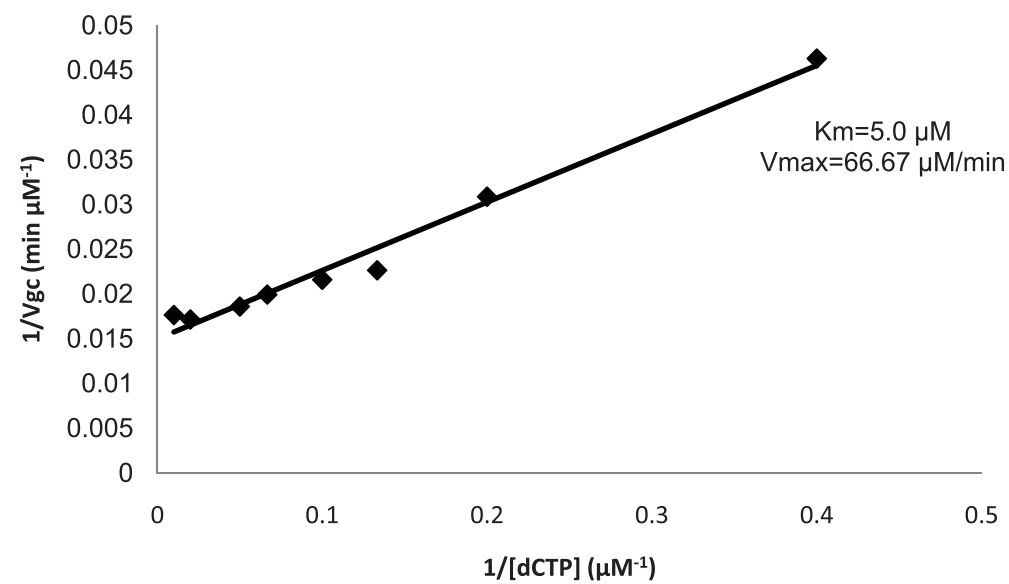

Supplement: Supplementary file 1 — Supplementary Material includes a representative steady state kinetic data analysis and a comparison of the sequences of MsDpo4 homologs from Mycobacteria. [file 285481.f1.pdf]
